# Near telomere-to-telomere genome assembly of the blackspot tuskfish (*Choerodon schoenleinii*)

**DOI:** 10.1038/s41597-025-04893-1

**Published:** 2025-03-31

**Authors:** Zhongdian Dong, Jiahao Gao, Yanfei Zhao, Jin Gao, Yusong Guo, Zhongduo Wang, Ning Zhang

**Affiliations:** 1 Key Laboratory of Aquaculture in the South China Sea for Aquatic Economic Animals of Guangdong Higher Education Institutes, College of Fishery, Guangdong Ocean University, Zhanjiang, 524088 China; 2 Agro-Tech Extension Center of Guangdong Province, Guangzhou, 510000 China; 3 Hainan Academy of Ocean and Fisheries Sciences, Haikou, 571126 China; 4 Guangdong Provincial Key Laboratory of Aquatic Animal Disease Control and Healthy Culture, College of Fishery, Guangdong Ocean University, Zhanjiang, 524088 China

**Keywords:** Genome, Genomics

## Abstract

*Choerodon schoenleinii*, commonly known as the blackspot tuskfish, widely recognized for its vibrant coloration, unique small black spot on the dorsal fin, and high value in both ornamental and culinary markets. Here, we report a high-quality near telomere-to-telomere (T2T) genome assembly of *C. schoenleinii*, generated using PacBio HiFi and Hi-C technologies. The assembly spans 865.99 Mb, achieving chromosome-level resolution with 24 chromosomes anchored. Notably, telomeres were identified at both ends of 23 chromosomes, with 14 being completely gapless and only 12 gaps detected across the remaining nine. A total of 24,524 protein-coding genes were annotated, with 96.25% assigned functional annotations. The assembly quality was validated with a BUSCO score of 99.80%. The gene annotation was further evaluated using OMArk, with 23,590 proteins (96.19%) consistent with the Clade Teleostei, highlighting the high-quality and taxonomic relevance of the gene set. This reference genome provides a valuable resource for advancing research in the genetics, evolutionary biology, conservation, and breeding of *C. schoenleinii*, a species currently listed as “Near Threatened” by the IUCN.

## Background & Summary

The genus *Choerodon*, belonging to the family Labridae, includes approximately 24 species, widely recognized for their vibrant coloration and unique dental morphology^1^. Among them, *Choerodon schoenleinii*, commonly referred to as the blackspot tuskfish, hereafter referred to as the tuskfish, is the largest species in the genus. Characterized by a small black spot on its dorsal fin, the tuskfish can grow up to one meter in length and weigh as much as 15.5 kilograms, making it ecologically and economically significant. This species inhabits sandy or weedy areas near lagoons and seaward reefs, where it primarily preys on hard-shelled organisms such as crustaceans, mollusks, and sea urchins^2,3^. Notably, observations have shown that individuals of this species can also utilize tools during feeding^4^. It exhibits protogynous hermaphroditism, transitioning from female to male as it matures, a process influenced by body size, age, and environmental factors^5–7^.

The tuskfish is predominantly found in the South China Sea, the coastal waters of Indonesia, and northern Australia. However, due to overfishing and marine environmental degradation, wild populations have experienced a sharp decline, leading to its classification as “Near Threatened” by the International Union for Conservation of Nature and Natural Resources (IUCN)^8,9^. To address this issue, comprehensive surveys of its wild germplasm resources and the development of artificial domestication and breeding techniques are urgently needed^10^.

In this study, we present a high-quality near telomere-to-telomere (T2T) genome assembly of the tuskfish, constructed using PacBio HiFi data at ~100× and Hi-C data at ~135×. The genome assembly spans 865.99 Mb and 24 chromosome-level scaffolds, of which 14 are gapless assemblies with terminal telomeric repeats. Notably, telomeres were resolved at both ends of 23 chromosomes, demonstrating high assembly completeness. A total of 24,524 protein-coding genes were annotated, with 96.25% assigned functional annotations. This dataset provides a valuable resource for genetic and evolutionary research, as well as for conservation and breeding programs aimed at restoring natural populations and promoting sustainable aquaculture production.

## Methods

### Sample collection and DNA extraction

Genomic DNA and total RNA were extracted from muscle and other tissues of a single *C. schoenleinii* specimen (female, body length: 24 cm, weight: 296 g) collected from the waters surrounding the Qizhou Archipelago. DNA was extracted using the Blood & Tissue DNA Kit (Qiagen 69504), while RNA was extracted with TRIzol reagent (Invitrogen) following the manufacturer’s protocols. DNA and RNA integrity were assessed using gel electrophoresis and an Agilent 2100 Bioanalyzer (Agilent Technologies), and purity and concentration were measured with a NanoDrop 2000 spectrophotometer (Thermo Fisher Scientific). High-quality DNA and RNA were used for library preparation and sequencing.

### Library construction and sequencing

For whole-genome sequencing (WGS), genomic DNA was fragmented into ~350 bp fragments using a Covaris ultrasonicator. After end-repair, adapter ligation, single-strand separation, and circularization, the library was amplified by rolling circle amplification (RCA) to generate DNA nanoballs (DNBs). Qualified DNB libraries were sequenced on the DNBSEQ platform, generating 143.70 Gb of data (~166×) (Table 1).

**Table 1:** Statistics of the sequencing data.

Library type	Platform	Tissue	Data size (Gb)	Average depth (×)
PacBio SMRT	PacBio REVIO	Muscle	86.54	100
Hi-C	Illumina Novaseq 6000	Muscle	117.52	135
WGS	DNBSEQ	Muscle	143.70	166
RNA-Seq	Illumina Novaseq 6000	Brain	6.13	—
RNA-Seq	Illumina Novaseq 6000	Gill	6.70	—
RNA-Seq	Illumina Novaseq 6000	Gut	6.71	—
RNA-Seq	Illumina Novaseq 6000	Heart	6.74	—
RNA-Seq	Illumina Novaseq 6000	Kidney	6.75	—
RNA-Seq	Illumina Novaseq 6000	Liver	6.74	—
RNA-Seq	Illumina Novaseq 6000	Muscle	6.72	—
RNA-Seq	Illumina Novaseq 6000	Ovary	6.72	—
RNA-Seq	Illumina Novaseq 6000	Skin	6.73	—
RNA-Seq	Illumina Novaseq 6000	Spleen	6.72	—

For PacBio HiFi sequencing, genomic DNA was used to construct a SMRTbell library with the SMRTbell Express Template Prep Kit 2.0. The library was sequenced on the PacBio REVIO system, producing 86.54 Gb of HiFi data (~100×) (Table 1).

For Hi-C sequencing, muscle tissue was fixed with 2% formaldehyde to cross-link DNA and proteins. Cross-linked DNA was digested with MboI, ligated with biotin-labeled adapters, circularized, fragmented, and enriched by biotin pull-down. Size-selected DNA was used to construct Hi-C libraries, which were sequenced on the DNBSEQ platform, producing 117.52 Gb data (~135×) (Table 1).

For transcriptome sequencing, RNA from 10 tissues, including brain, gill, gut, heart, kidney, liver, muscle, ovary, skin, and spleen, was used to construct transcriptome libraries. Poly-A mRNA was enriched using magnetic oligo(dT) beads, fragmented, and reverse-transcribed into cDNA. Libraries were prepared with adapter ligation and sequenced on the Illumina NovaSeq 6000 platform, yielding 66.66 Gb transcriptome data (Table 1).

### Genome survey and assembly

**Fig. 1:**
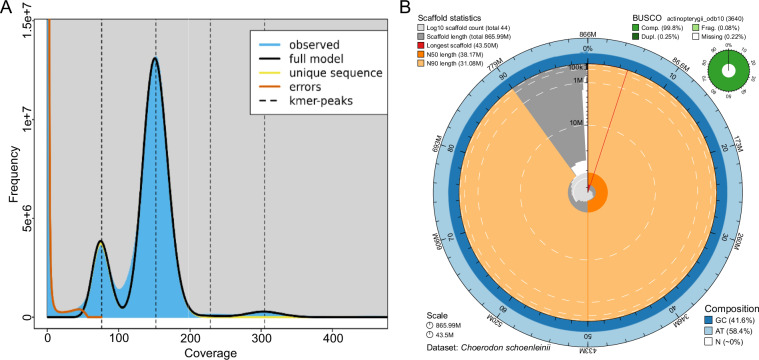
Summary of the blackspot tuskfish genome assembly and quality assessment. (**A)** GenomeScope *k*-mer analysis (*k* = 19) of whole-genome sequencing reads. **(B)** Snail plot of assembly statistics. The plot displays, from the center outwards: log-scaled scaffold count (purple spiral), scaffold length distribution (dark gray, longest scaffold in red), N50 (orange) and N90 (light orange) lengths, and GC/AT content (blue/light blue rings). The actinopterygii BUSCO score is shown in the upper right.

A genome survey was conducted prior to assembly to assess the basic characteristics of the tuskfish genome. Short reads generated by the DNBSEQ platform were used for k-mer analysis, estimating the genome size at 815.81 Mb (Fig. 1A).

**Fig. 2:**
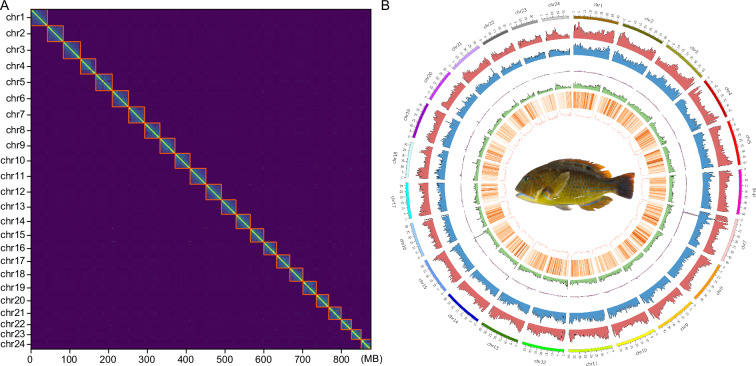
Hi-C contact maps and genomic feature overview of the blackspot tuskfish genome. (**A)** Interchromosomal interaction matrix. A heatmap representing Hi-C interaction frequencies (100-kb bins) across the 24 blackspot tuskfish chromosomes (chr1-chr24) **(B)** Circos plot summarizing genomic features. Concentric rings display, from outermost to innermost: (1) Chromosome ideograms; (2) DNA transposon density; 1 (3) LINEs density; 1 (4) SINEs density; (5) LTRs density; 1 (6) Gene density; (7) GC content. 1 A central image depicts a blackspot tuskfish. All feature densities are presented along the chromosomes.

For genome assembly, HiFi reads were first assembled into a draft genome using Hifiasm (v0.20.0)^11^, resulting in a highly contiguous assembly with a total size of 879.13 Mb. To improve the quality of the assembly, redundant sequences and haplotigs were removed using purge_haplotigs^12^ and kmerDedup^13^, reducing the genome size to 865.99 Mb with contig N50 38.17 Mb (Fig. 1B). Hi-C sequencing data were then integrated using HapHic (v1.0.6)^14^ and further refined using Juicer (v1.6)^15^ to anchor the contigs to 24 chromosomes (Fig. 2A), which is consistent with the number of chromosomes already demonstrated in some closely related species^16–18^.

To further enhance genome quality, polishing was performed using NextPolish (v1.4.1; https://github.com/Nextomics/NextPolish), significantly reducing assembly errors. The final assembly consisted of 14 completely gapless chromosomes, with gaps closed and telomeres detected at both ends of 23 chromosomes using quarTeT (v1.23)^19^ (Table 2).

**Table 2:** Assembly statistics of chromosomes.

Chromosome number	Length (Mb)	Number of gaps	Number of telomeres
chr1	41.62	1	2
chr2	40.11	0	2
chr3	43.50	0	2
chr4	38.80	0	2
chr5	41.57	1	2
chr6	41.37	1	2
chr7	40.86	1	2
chr8	38.69	0	2
chr9	38.17	3	2
chr10	37.54	0	2
chr11	40.34	0	2
chr12	38.98	0	2
chr13	37.01	1	2
chr14	36.24	0	2
chr15	33.77	1	2
chr16	32.23	0	2
chr17	33.04	1	1
chr18	33.29	0	2
chr19	33.87	0	2
chr20	31.08	0	2
chr21	31.51	0	2
chr22	25.74	0	2
chr23	24.57	1	2
chr24	24.72	1	2
Unplaced^a^	7.33		

^a^Unplaced: Sequences that could not be anchored to any known chromosome.

The chromosome numbering (chr1 to chr24) follows the order established in the published genome of *Notolabrus celidotus* (GCF_009762535.1) on NCBI.

### Genome annotation

Transposable element (TE) annotation was performed using HiTE^20^, which identified and masked repetitive elements across the genome. A total of 32.07% of the genome was annotated as repetitive sequences, consistent with the genome survey results (Fig. 2B; Table 3).

**Table 3:** Statistic results of different types of annotated repeat content.

Type	Length (bp)	% of genome
DNA	136,347,261	15.75
LINE	87,187,153	10.07
SINE	5,697,663	0.66
LTR	42,444,577	4.90
Other	118,586	0.01
Unknown	5,871,707	0.68
Total	255,401,065	32.07

Protein-coding gene annotation combined *de novo* prediction, homology-based annotation, and transcriptome-based strategies. *De novo* gene prediction was performed using Augustus (v3.5.0)^21^ and GALBA (v1.0.11)^22^, while homology-based annotation employed miniport (v0.13)^23^ to align the genome against protein sequences from closely related species, inculding *Cheilinus undulatus*, *Labrus bergylta*, and *Notolabrus celidotus*, generating homology-based gene models. Transcriptome data were integrated with homology-based annotations using EGAPx (v0.3.1-alpha; https://github.com/ncbi/egapx), which prepared input data for downstream integration. The final gene models were produced by integrating results from all annotation strategies using EvidenceModeler (EVM; v2.10)^24^ and further refined using the PASApipeline (v2.5.3)^25^, resulting in the annotation of 24,524 protein-coding genes (Table 4).

**Table 4:** Genome function annotation result.

	Number	Percentage (%)
Total	24,524	100
NR	22,780	92.89
EggNOG	23,109	94.23
KEGG	18,804	76.68
Kofam	15,426	62.90
SwissProt	18,019	73.48
Overall	23,604	96.25

Functional annotation of the protein-coding genes was conducted by aligning protein sequences against multiple databases, including KEGG, NR, SwissProt, Kofam, and EggNOG, using diamond. A total of 96.25% of the protein-coding genes were functionally annotated, providing valuable insights into the biological roles of the identified genes (Table 4).

## Data Records

The genome assembly data is available in GenBank under the accession number JBKFGB000000000.1^26^.

The raw sequencing data of blackspot tuskfish transcriptome, PacBio HiFi, Hi-C and WGS have been deposited into the National Center for Biotechnology Information (NCBI) with the accession number PRJNA1204159^27^. The genome assembly data, genome annotation files, gene CDS, and protein data have been submitted to Figshare^28^.

## Technical Validation

Genome assembly was evaluated with a Benchmarking Universal Single-Copy Orthologs (BUSCO), achieving 99.8% completeness, confirming the genome’s high completeness. Inspector (v1.3)^29^ calculated a QV of 48.54, reflecting high base-level accuracy, while GCI (v1.0)^30^ yielded a value of 43.681, highlighting the structural continuity of the assembly. CRAQ (v1.0.9)^31^ further assessed assembly accuracy, reporting AQI metrics of 95.71 (R-AQI) and 99.88 (S-AQI), confirming high assembly quality. Additionally, 14 chromosomes were completely gapless, and telomeres were detected at both ends of 23 chromosomes, demonstrating near telomere-to-telomere assembly quality.

Annotation quality was validated with BUSCO, which reported 98.5% completeness for the gene sets. OMArk (v.0.3.0)^32^ showed that 96.19% (23,590 proteins) of the annotated genes were consistent with the Clade Teleostei, and 96.25% of the genes were functionally annotated across databases such as KEGG, NR, and SwissProt, indicating high functional and structural completeness.

## Data Availability

All software used in this study is in the public domain, with parameters described in Methods and this section. If no detailed parameters were mentioned for the software, default parameters were used according to the software introduction.
